# Early gene expression changes with rush immunotherapy

**DOI:** 10.1186/1476-7961-9-12

**Published:** 2011-09-30

**Authors:** Laurie S Davis, Sumit Bhutani, Sherry Ridz Barnett, David A Khan

**Affiliations:** 1 Department of Internal Medicine, Division of Rheumatic Diseases, University of Texas Southwestern Medical Center, Dallas, TX, 75390-8884, USA; 2 Department of Internal Medicine, Division of Allergy and Immunology, University of Texas Southwestern Medical Center, Dallas, TX, 75390-8859, USA

**Keywords:** Rush immunotherapy, allergy, gene expression

## Abstract

**Background:**

To examine whether whole genome expression profiling could reveal changes in mRNA expression of peripheral blood mononuclear cells (PBMC) from allergic patients undergoing rush immunotherapy (RIT) that might be manifest within the first few months of treatment.

**Methods:**

For this study, PBMC from three allergic patients undergoing RIT were assessed at four timepoints: prior to RIT, at 1 week and 7 week post-RIT, during build-up and at 4 months, after establishment of a maintenance dose. PBMC mRNA gene expression changes over time were determined by oligonucleotide microarrays using the Illumina Human-6 BeadChip Platform, which simultaneously interrogates expression profiles of > 47,000 transcripts. Differentially expressed genes were identified using well-established statistical analysis for microarrays. In addition, we analyzed peripheral blood basophil high-affinity IgE receptor (Fc epsilon RI) expression and T-regulatory cell frequency as detected by expression of CD3^+^CD4^+^CD25bright cells at each timepoint using flow cytometry.

**Results:**

In comparing the initial 2 timepoints with the final 2 timepoints and analyzing for genes with ≥1.5-fold expression change (p less than or equal to 0.05, BH-FDR), we identified 507 transcripts. At a 2-fold change (p less than or equal to 0.05, BH-FDR), we found 44 transcripts. Of these, 28 were up-regulated and 16 were down-regulated genes. From these datasets, we have identified changes in immunologically relevant genes from both the innate and adaptive response with upregulation of expressed genes for molecules including IL-1β, IL-8, CD40L, BTK and BCL6. At the 4 month timepoint, we noted a downward trend in Fc epsilon RI expression in each of the three patients and increased allergen-specific IgG4 levels. No change was seen in the frequency of peripheral T-regulatory cells expressed over the four timepoints.

**Conclusions:**

We observed significant changes in gene expression early in peripheral blood samples from allergic patients undergoing RIT. Moreover, serum levels for allergen specific IgG4 also increased over the course of treatment. These studies suggest that RIT induces rapid and dynamic alterations in both innate and adaptive immunity which can be observed in the periphery of allergic patients. These alterations could be directly related to the therapeutic shift in the allergen-specific class of immunoglobulin.

## Introduction

While a number of immunologic changes occur with allergen immunotherapy (IT), the relationship of these various changes to the overall effectiveness of IT is unclear. There are several immunologic changes seen with IT, including: decreases in allergen-specific IgE, increases in IgG4 "blocking" antibodies, suppression of the classic TH2 cytokines with a rise in TH1 cytokine expression, and an increase in the frequency of T-regulatory cell populations [1-3].

Rush IT (RIT) is a form of accelerated IT where patients undergo a series of dose escalating injections over a single or two-day period in order to achieve a maintenance dose earlier than with conventional IT. This form of IT has been proven to be both safe and effective [4,5].

Genome-wide transcriptional profiling has been shown to be a useful tool to identify and classify human diseases. Gene expression profiling has been used to identify whether patients will respond to certain drug therapies, to assess disease response to therapy, and to predict unwanted drug side-effects [6,7]. While gene expression changes have been used for a number of years to study autoimmune diseases and cancer, less is known about the changes seen with allergic diseases [8-10].

Allergy related genes have been identified through the use of gene profiling, but little is known about gene expression changes that occur with IT [11]. Liu et al. conducted a study to evaluate gene expression changes in patients undergoing IT. The goal of the study was to identify a unique gene profile in RIT patients compared to healthy controls and those with autoimmune diseases. The study followed 4 patients on RIT using a limited cDNA microarray of 4100 genes. After 4 months on IT, the authors identified under-expressed genes encoding apoptosis-related proteins, and over-expressed transcripts encoding proteins involved in stress response and signal transduction [12]. Another more recent study evaluated patients on venom IT and identified osteopontin as a potential biomarker [13].

In this study, our primary outcome was to examine immunologic changes in a group of patients undergoing RIT to multiple inhalant allergens. In addition to genome-wide transcriptional profiling, we assessed whether RIT would have an effect on basophil FcεRI expression as this has not been looked at previously. With anti-IgE therapy, surface FcεRI expression is known to decrease in correlation with diminished free IgE levels[14]. We hypothesized that changes in gene expression would be evident early in the course of RIT. In addition, we monitored immunologic parameters, including the frequency of CD3^+^CD4^+^CD25^bright ^T-regulatory cells, allergen-specific IgE and IgG4 and basophil FcεRI expression.

## Methods

### Patient Selection

Eligible patients for this pilot study were recruited from our university hospital allergy clinic. These studies were approved by the Institutional Review Board at UT Southwestern Medical Center. The patient blood samples were collected after obtaining written informed consent from the study subjects. For this study, we enrolled 3 patients undergoing RIT to multiple aeroallergens. In order to qualify, patients had to demonstrate skin test positivity to one of 4 common major aeroallergens: bermuda grass, ragweed, and *Dermatophagoides *(*D*.) *pteronyssinus*, or *D. farinae *house dust mites. Only adult patients who had ≥ a 5 mm wheal on prick testing to at least 1 of the study allergens were eligible. Exclusion criteria included those patients who had previously received IT, or were on chronic corticosteroids, were pregnant, used β-blocker medications, or had uncontrolled asthma.

Patients underwent RIT per our standard protocol: a series of injections over a three hour period to achieve a 1:10 dilution of allergen concentrate with subsequent build-up to undiluted allergen concentrate over the next several weeks [15]. Our standard RIT protocol is outlined in Table 1. The Rhinitis Symptom Severity Assessment instrument was obtained prior to initiation and after 4 months of RIT (Table 2). This rhinitis symptom severity questionnaire is a visual analog scale that scores nasal (e.g. sneezing, runny nose) and non-nasal (e.g. chronic cough, eye symptoms) symptoms. Each symptom is scored between a one (none to an occasional episode) to seven (unbearably severe) [16]. Appropriate samples were collected from patients for each of the assays at various timepoints as shown in Table 3.

**Table 1:** Rush Immunotherapy Protocol

Injection Number	Time (Min)	Concentration (Volume:Volume)	Volume (ml)
1	0	1:10,000	0.3
2	30	1:1,000	0.3
3	60	1:100	0.1
4	90	1:100	0.3
5	120	1:10	0.1
6	180	1:10	0.2

Following the first day of RIT, the patient receives a weekly injection of concentrate extract as follows: 0.02, 0.05, 0.1, 0.2, 0.3, 0.4, 0.5 ml. Injections of 0.5 ml are given once at 2 weeks, then once at 3 weeks, then at monthly intervals.

**Table 2:** Patient Demographics and Symptom Assessment

			Skin Test Positivity*				Avg. Nasal SymptomScore**	
Patient	Age	Race	Bermuda	Ragweed	D. Pteryon	D.Farinae	Baseline	4 mos. Post-RIT
1	56	Caucasian	^**+**^	^**+**^			4.0	1.7
2	32	Caucasian	^**+**^	^**+**^	^**+**^	^**+**^	5.1	2.9
3	28	Indian	^**+**^	^**+**^	^**+**^	^**+**^	4.0	3.7

* + = greater than or equal to 5 mm on skin prick testing

** *Rhinitis Symptom Severity Score*, Range 1 (no symptoms) to 7 (unbearably severe symptoms)

**Table 3:** Timetable for Collection of RIT Assessment and Samples

	Timepoints				
	Pre RIT	1 week	7 week	16 week	52 week
Rhinitis Symptom Severity Assessment	X			X	
Oligonucleotide Microarray	X	X	X	X	
Basophil FcεRI expression	X	X	X	X	X
Treg population	X	X	X	X	X
Allergen specific IgE/IgG4	X			X	X

Patient samples were collected for the analyses shown over the course of the 52 week study. Routine laboratory blood cell counts were carried out at each visit and are not shown.

### RNA Extraction and Microarray Expression Profiling

Peripheral blood mononuclear cells (PBMC) from RIT patients were used for the various assays. Of note, no significant differences were observed in routine laboratory blood cell counts and leukocyte differential values for individual samples collected at the different timepoints. To ensure minimum *in vitro *impact on the activation status of the cells, we employed a modified gradient separation. PBMC were immediately isolated by a rapid Ficoll-Hypaque centrifugation for 15 min at 800 × g. Cells were washed in PBS and cell numbers were counted. Total cellular RNA was extracted from 10 million PBMC with Trizol (Invitrogen) and immediately stored at -80°C until further purified using the *Qiagen RNeasy Mini Kit*. RNA integrity and quantity was confirmed by bioanalyzer. All cRNA probes, oligonucleotide microarray manipulations, and scanning of the arrays were carried out by our genomics and microarray core facility adhering to stringent quality control criteria and data processing that are detailed at the core website (http://microarray.swmed.edu/) which also includes numerous prior publications. PBMC mRNA gene expression changes over time were determined by oligonucleotide microarrays using the *Illumina Human-6 BeadChip *Platform. Of the 47,289 probe sets, 33,458 transcripts had flag calls of present. We assessed the statistical significance of differentially expressed genes by standard methods. The data was normalized for all probe sets to the median of all samples and the data was filtered for differential expression using the *GeneSpring 7.3.1 Analysis Platform *(Agilent Technologies). For observed gene expression changes over time with RIT, we compared the initial 2 timepoints (pre-RIT and 1 week after the first RIT treatment) with the final 2 timepoints (7 week and at 16 week post initiation of RIT). The comparisons were subjected to statistical analysis as detailed in the text. Annotation and functional assignments were assessed with Ingenuity Pathways Analysis (IPA) Software (Ingenuity, Inc.). For analysis purposes the IPA software assigns gene names (based on each probe, indicated in parentheses in the text) to all mRNAs and associated molecules, however, in many cases the gene and protein names are identical. Microarray data is available through the NCBI GEO database (GSE29521).

### Flow Cytometry Analyses

Basophils were identified as Lin-1^-^, HLA-DR^- ^and CD123^+ ^using BD Pharmingen antibodies. The BD Lineage cocktail (Lin-1) contains antibodies to CD3, CD14, CD16, CD19, CD20 and CD56. Basophil high-affinity receptor (FcεRI) expression was detected with an antibody to FcεRI-alpha (antibody clone AER-37 obtained from eBioscience). T regulatory cells (CD3^+^CD4^+^CD25^bright^) were identified at each timepoint (Table 3) using directly-labeled antibodies (BD Pharmingen) and detected by flow cytometry (BD FACSCalibur).

### Allergen-Specific Antibody Determinations

RIT patient serum samples were immediately aliquoted and stored at -80°C. Samples were shipped on dry ice to the Johns Hopkins Dermatology, Allergy and Clinical Immunology. Allergen specific IgE levels, reported as kU[A]/L (kilounits of allergen-specific IgE per liter), were obtained using an ImmunoCAP250 device (Phadia Uppsala, Sweden). Allergen specific IgG4 levels, reported as mg [A]/L (milligram allergen-specific IgG4 per liter), were obtained using a UniCap100 device (Phadia Uppsala, Sweden). These results were analyzed courtesy of Dr. Robert Hamilton of the Johns Hopkins Dermatology, Allergy and Clinical Immunology Reference Laboratory, which is a CLIA-88 certified clinical laboratory. Statistical analysis of the results was carried out using a nonparametric paired t-test (GraphPad Prism 5).

## Results

### Patient Demographics and Symptoms

Patient demographics are shown in Table 2. All three patients were sensitive to bermuda grass and ragweed, and two of the three patients were also sensitive to the two dust mite species. All three patients demonstrated improvement in their symptoms with two of three patients having a large improvement in their allergic symptomatology. None of the three patients were asthmatic.

### Allergen Specific IgG

**Figure 1:**
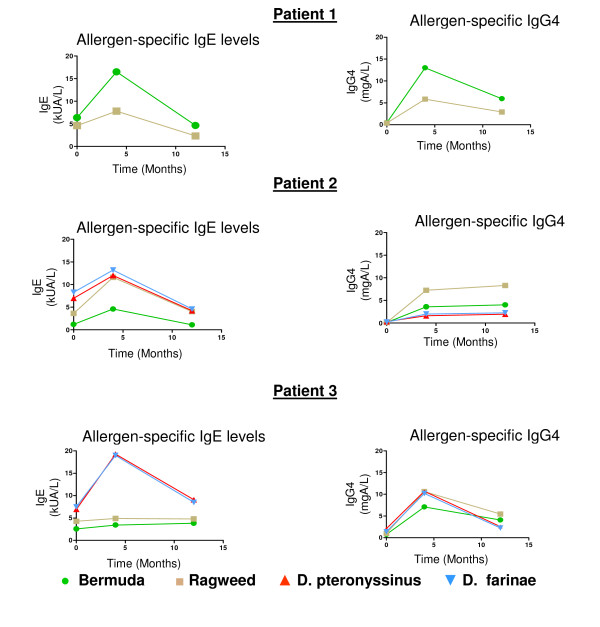
**Allergen specific IgE and IgG4 from RIT patients evaluated over time**. Serum from each of the RIT patients was collected at various timepoints, pre-RIT, at the 16 week (4 month) visit before RIT was administered and at the 52 week (12 month) visit before treatment. Allergen specific IgE levels (kU [A]/L) were assessed by the ImmunoCAP250 method. Allergen specific IgG4 levels (mg [A]/L) were assessed by the UniCap100 method. Aeroallergens included Bermuda grass (green circle), Ragweed (brown square), *D. pteronyssinus *(red up-triangle) and *D. farinea *(blue down-triangle).

Allergen specific IgE and IgG4 levels are shown in Figure 1. We analyzed these antibody levels at three timepoints: pre-RIT, at 16 weeks (4 months)maintenance dose, and after a one year follow-up. As with conventional IT, there was an initial rise in allergen specific IgE with a downward trend at 1 year [17]. All patients had an increase in allergen specific IgG4 at 1 year following RIT compared to baseline (ranging from a 1.2 to a 63.8 fold increase). Specifically, the combined pre-RIT Bermuda IgG4 levels were 0.43 ± 0.31(mean mg [A]/L ± SD) compared to one year levels of 4.68 ± 1.08 (p < 0.05), the pre-RIT Ragweed IgG4 levels were 0.50 ± 0.43 versus one year levels of 5.55 ± 2.69 and the combined dust mite species allergens IgG4 levels were 1.0 ± 0.24 versus one year levels of 2.25 ± 0.24 (p < 0.05). Thus, these studies demonstrate that RIT was effective at enhancing allergen specific IgG4 levels that could be detected in the serum.

### Transcriptional Profiles with RIT

We carried out whole genome expression profiling on patient PBMC. We compared the initial 2 timepoints, pre-RIT and 1 week after RIT, with the final 2 timepoints, 7 weeks and at 16 weeks (or 4 months) post-RIT. To minimize the inclusion of genes not related to RIT, we subjected the dataset to analysis by a Welch *t*-test combined with a Benjamini and Hochberg False Discovery Rate (BH-FDR) multiple testing correction with a FDR of 5% to control for transcripts that might appear by chance. A number of transcripts (507) were differentially expressed at ≥1.5 fold (p < 0.05, BH-FDR). We examined the cellular localization and biological processes for the molecules represented by these transcripts by IPA analysis (Additional file 1, Table S1). This global analysis revealed that the majority of the transcripts were for cytoplasmic molecules (32%) followed by molecules localized to the nucleus (26%), the plasma membrane (12%), and the extracellular space (4%). The localization of the molecules represented by the remaining transcripts (26%) were unmapped. Two notable molecules that directly regulate B cell function include Bruton's tyrosine kinase (BTK) and B-cell CLL/lymphoma 6 (BCL-6). Overall, the proteins from these transcripts fell into diverse functional categories including: enzymes (80), transcriptional regulators (53), transporters (35), kinases (28), phosphatases (19), peptidases (14), transmembrane receptors (14), cytokines/chemokines (7), translational regulators (4), ion channels (3), G-protein coupled receptors (3) and ligand-dependent nuclear receptors (1). The molecular functions for the proteins produced by the remaining transcripts have yet to be identified.

**Figure 2:**
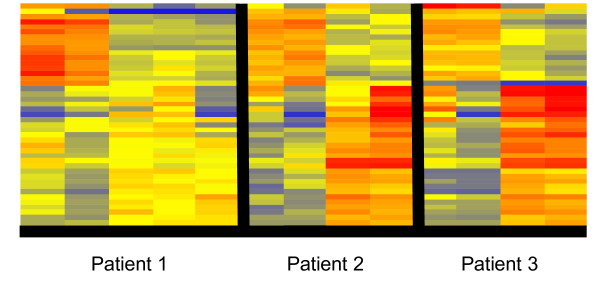
**Heat maps of differentially expressed transcripts (≥2-fold change, p ≤ 0.05) after hierarchical clustering was performed to visualize up and down-regulated transcripts**. The dendrogram shown compares pre-RIT and 1 week timepoints (columns 1 and 2 for each patient) versus 7 and 16 week timepoints (columns 3 and 4 for each patient). Included is a 20 week PBMC sample which was collected and processed for microarray for the first RIT patient only (patient 1, column 5). Overall there were 28 up-regulated transcripts and 16 down-regulated transcripts. In the dendrogram red indicates increased expression relative to the median of all samples, blue indicates decreased expression relative to the median of all samples and yellow indicates the median of all samples.

**Figure 3:**
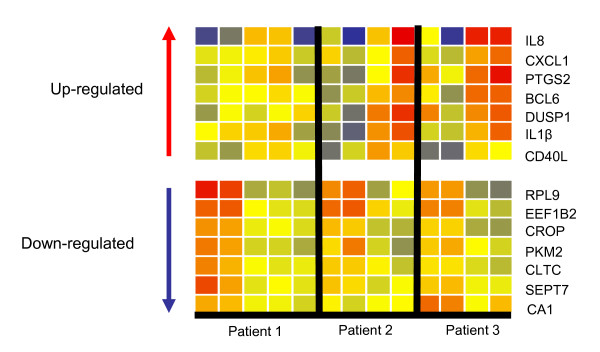
**Select up-regulated and down-regulated transcripts**. The dendrogram shown highlights the variable expression that was observed at different timepoints for individual patients although collectively many transcripts were coordinately up or down-regulated over the course of the treatment. As above, the heat map is comparing pre-RIT and 1 week timepoints (columns 1 and 2 for each patient) versus 7 and 16 week timepoints (columns 3 and 4 for each patient). The 20 week PBMC sample for the first RIT patient (patient 1, column 5) is also shown. As in Figure 2, red indicates increased expression relative to the median of all samples, blue indicates decreased expression relative to the median of all samples and yellow indicates the median of all samples.

At a ≥2 fold differential expression, 44 transcripts (p < 0.05, BH-FDR) were detected. Of these, 28 were up-regulated and 16 were down-regulated. These transcripts were subjected to unsupervised hierarchical clustering to group the samples on the basis of similarity and visualized as dendrograms. A significant alteration in the transcriptional profile was readily detected between the pre and 1 week post RIT sample as compared to samples collected at or after 7 weeks (Figure 2). Of note, the first RIT patient also had a 20 week (5 month) PBMC sample which was processed for microarray as shown in Figures 2 and 3 (patient 1, column 5), however, the data for this patient was excluded from the remainder of the analysis and the 20 week microarray results are shown only on the dendrograms. A select list of transcripts demonstrating the significant changes post-RIT is shown in Figure 3. Of note IL-1β, IL-8, and CD40L were significantly up-regulated by this analysis. In addition, other molecules known to regulate both innate and adaptive immune responses including the chemokine CXCL1 and the dual specificity phosphatase 1, DUSP1, were up-regulated during RIT.

### Basophil FcεRI Expression after RIT

**Figure 4:**
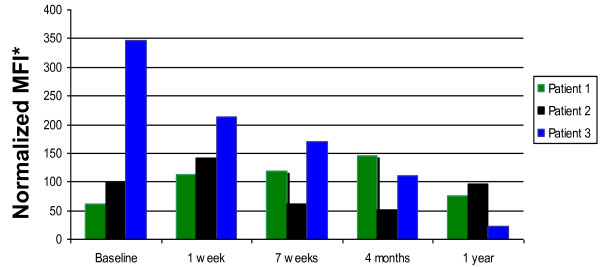
**Basophil FcεRI expression for RIT patients over time**. Basophils were identified as Lineage-1^-^, HLA-DR^- ^and CD123^+ ^(BD antibodies). Basophil high-affinity receptor (FcεRI) expression was detected with an antibody to FcεRI-alpha (eBioscience) and detected by flow cytometry (BD FACSCalibur). Data are expressed as the normalized mean fluorescence intensity (MFI) of the RIT patient sample relative to a control in the same experiment for each of the timepoints indicated including pre-RIT, 1 week post initiation of RIT and before treatment was administered (post-RIT), 7 weeks post-RIT, 16 weeks post-RIT (4 months) and 52 weeks (1 year) post-RIT. Results for individual patients are presented as blue, black and green bars.

Basophil FcεRI expression before and during the first year of RIT is depicted in Figure 4. Interestingly, patient 3 had a steady decline in basophil FcεRI expression over the year following RIT. Patient 3 also had the highest baseline expression of basophil FcεRI expression. In contrast, no consistent trend in basophil FcεRI expression was observed in patients 1 and 2.

### CD4^+ ^T-regulatory populations after RIT

In defining T-regulatory cells as CD3^+^CD4^+^CD25^bright ^cells, all three patients had a reduction in the percentage of T regulatory cells from baseline to 16 weeks. However, at one year follow-up, only one patient exhibited a persistent reduction in the frequency of circulating T-regulatory cells (data not shown).

## Discussion

Our results indicate that gene expression changes can be observed early in the course of immunotherapy in PBMC obtained from patients on a rush protocol. In fact, we were able to identify several gene transcripts that appeared to change as early as 1 week following RIT. In order to be able to draw conclusions from this pilot study, we grouped our samples from two timepoints. We separately compared early timepoints (baseline and 1-week post-RIT) with later timepoints (7 and 16 weeks post-RIT) when patients were near or on a maintenance dosage of IT. When analyzing the early versus later timepoints, we identified transcripts for well known molecules involved in the regulation of immune responses such as IL-8, IL-1β, CD40L, CXCL1, BCL-6, BTK and COX2 (*PTGS2 *gene) which were all up-regulated. Interestingly, IL-8 (CXCL8) demonstrated the greatest fold change of all up-regulated transcripts. The chemokine IL-8 is well known to play a role in the rapid mobilization of hematopoietic progenitor cells and to induce both systemic and local leukocyte migration [18]. Moreover, IL-8, IL-1β, and CXCL1 are produced by monocytes and activate target cells during inflammatory responses [18]. Whereas IL-8 was strongly induced, CXCL1 was modestly up-regulated by 1.5 fold change. Although the receptors for IL-8 and CXCL1 were also up-regulated, CXCR1 by 1.3 fold change and CXCR2 by 1.8 fold change, they did not achieve statistical significance, however, these results suggest that this pathway demonstrated heightened activity in peripheral PBMC at 7 weeks and 16 weeks post initiation of RIT.

BCL-6 and Cyclooxygenase 2 (COX 2) act to promote leukocyte survival [19,20]. Importantly, B cell differentiation into plasma cells is regulated by CD40 Ligand (CD40L or CD154) expressed by activated T cells. BCL-6 affects both innate and adaptive responses as BCL-6 is a transcriptional repressor in dendritic cells and more recently BCL-6 has been demonstrated to be a master regulator for T follicular helper cell development in addition to its well known crucial role in germinal B cell formation [20]. Thus expression of these transcripts likely reflect the role of these molecules in the generation of allergen-specific IgG4 secreting B cell populations. Interestingly, both DUSP1 (dual specificity phosphatase 1) and COX2 are known regulators of inflammatory responses and both have been associated with autoimmunity. Therefore these gene expression changes appear to reflect the pro-inflammatory process that is occurring at early timepoints during successful RIT [21-23].

A survey of OMIM suggests that there is little information regarding the proteins of the most statistically significant, down-regulated transcripts with known function observed early in the leukocyte response to RIT and that there is no clear correlation with pro-inflammatory responses. Several molecules identified by these transcripts have been reported to be involved in transcriptional regulation including: Ribosomal Protein L9 (*RPL9*), Eukaryotic Translation Elongation Factor 1 beta-2 (*EEF1B2*), which is a major protein in eukaryotic cells that conducts the enzymatic delivery of aminoacyl tRNAs to the ribosome and Cisplatin Resistance-associated Overexpressed Protein (*CROP*) which is a molecule found in all cells and that is involved in RNA splicing [24-26]. Other down-regulated transcripts identified molecules including: Pyruvate Kinase, M2 (*PKM2*) which is a glycolytic enzyme involved in cellular metabolism, Clathrin, Heavy Polypeptide (*CLTC*) which is a major component of coated vesicles and coated pits and Cell Division Cycle 10 (*CDC10 *or *SEPT7*) which is a molecule that is part of a signaling pathway involved in the regulation of the DNA damage response to the cytoskeleton and Carbonic Anhydrase I (*CA1*) which is a zinc metalloenzyme which has been implicated in lymphocyte maturation. Further pathway analysis of an expanded lists of transcripts down-regulated in response to RIT might further our understanding of the mechanisms regulating the leukocyte response to therapy.

We interrogated the dataset for known allergy-related genes whose regulation might contribute to a therapeutic response. We found that transcripts related to both TH1 and TH2 responses were ≥1.5 fold up-regulated in leukocytes during RIT. These included transcripts for cytokine and chemokine receptors such as: *IFNGR1 *(CD119), *IFNGR2*, *IL13RA1*, *CCR3*, *IL2RB*, the TH2 transcription factor *GATA3*, the apoptosis-related receptor TRAIL (*TNFSF10*) and Caspase 3, an apoptosis-related cysteine protease in addition to cell surface molecules CD33, CD89, and CD53. Other transcripts of interest up-regulated by ≥1.4 fold included: TNF, *A20*, TRAF1, TRAF5, *ITGAM*, CD44, CD32 and CD107b which have been implicated in autoimmune and other diseases. Interestingly, the transitional B cell markers CD10 and BTK, were up-regulated by ≥1.7 fold suggesting ongoing alterations in peripheral B cell maturation. Finally, type I interferon-induced transcripts for the molecules *MX-1*, *IFIT2*, and *IRF1 *were up-regulated by ≥1.7 fold. Although these results are intriguing, the significance of these transcripts require confirmation with a larger sample size and further dissection of the immune response during RIT. Taken together, these finding suggest that RIT induces a rapid and potent pro-inflammatory TH1 response that results in enhanced IgG4 production by B cells to specific allergens.

Previous studies utilizing oligonucleotide microarrays to analyze patients with allergic rhinitis have focused primarily on comparing patients with an allergic phenotype to healthy controls. Heishi et al., analyzed PBMCs in atopic dermatitis patients and found 4 transcripts, (IFN-γ, TRAIL, ISGF-3, and defensin-1), through screening GeneChip and confirmatory PCR, that were significantly different in atopic dermatitis patients compared to healthy controls [27]. Benson et al. analyzed nasal mucosal biopsies of allergic rhinitis patients and used cDNA microarrays to focus on 32 transcripts thought to be relevant to mucosal inflammatory responses (e.g., cytokines, growth factors, eosinophil, and neutrophil granulae proteins) [28]. In comparing the allergic patients to healthy controls, the authors found only modest differences in the gene expressions of the 32 genes. More recently, Zhang et al. analyzed gene expression of chemokines and their receptors in the nasal mucosa AR patients compared to healthy controls [29]. These authors found upregulation of a majority of chemokines and chemokine receptors in AR patients.

The only study, to our knowledge, to analyze gene expression profiles in patients undergoing RIT was conducted by Liu et al. [12]. In that study, peripheral blood samples were obtained for analysis prior to RIT and at approximately 4 months when patients were on maintenance IT. Limitations in that study include that the analysis was performed at only 2 timepoints as well as the microarray dataset analyzed only 4100 genes. TGF-β was the only transcript that the authors identified with greater than a two-fold difference between before and after immunotherapy, but the difference did not reach statistical significance. In comparing our gene expression results with previous studies, our findings are unique. One strength of our study includes the fact that we performed gene expression profiles at several early timepoints during the course of RIT. By doing so, we found gene expression changes as early as 1 week after the initiation of RIT.

We analyzed the allergen specific antibodies, specifically IgE and IgG4, over the course of one year as a secondary outcome. As expected, the allergen specific IgE for each allergen peaked at the 16 week (4 month) interval before trending down. A previous RIT study following IgE and IgG4 levels sequentially for patients on birch IT found the peak IgE at 2 months before seeing a steady decline at several timepoints out to 1 year [30]. This same study found the allergen specific IgG4 levels to steadily increase over time up to 1 year of IT. In our patients, the allergen specific IgG4 levels were at the highest amounts at the 1 year timepoint only for patient 2. For patients 1 and 3, the allergen specific IgG4 levels peaked at the 4 month timepoint. If we had continued to follow the allergen specific IgE and IgG4 further over time, we predict a continued steady decline in the IgE levels with a rise in IgG4 levels.

While we did not expect major changes in basophil FcεRI expression, surprisingly, patient 3 showed a steady decline over time after RIT. Interestingly, patient 3 also had the highest baseline FcεRI expression. Since total IgE is not affected by IT, this suggests that perhaps other regulatory factors may be involved with FcεRI expression. We confirmed the validity of our FcεRI assay by assessing the known down-regulation of FcεRI expression after omalizumab therapy (Additional file 2, Figure S1). We analyzed peripheral blood samples from 2 asthmatic non-IT patients on chronic monthly omalizumab therapy and these samples exhibited a striking down-regulation in basophil FcεRI expression as compared to controls. To our knowledge, no studies have looked at FcεRI expression changes with IT. This may be important component to analyze in larger IT studies.

CD4^+ ^T-regulatory frequencies tended to be higher in 2 of 3 patients at the last timepoint. We did not observe a steady increase in the percent of T-regulatory cells that has been described with conventional IT [31]. Longer follow-up may be required to demonstrate more robust changes. In addition, intracellular staining for FoxP3 or gating on cells with low CD127 expression may more accurately discriminate the peripheral population of T-regulatory cells [32]. Alternatively, recent studies suggest that T-regulatory cells could be organ-specific and thus assessing frequencies in the periphery might not be optimal for a given response [33,34].

Our study had several limitations. First, because this was a pilot study, our small patient population is a limitation. However, by grouping the patient samples, we were able to strengthen the microarray analysis findings. In addition, we enrolled patients undergoing RIT to multiple allergens. This heterogeneity in allergens may have confounded some of our results but it is commonplace in our clinical practice, to see allergic patients with multiple sensitivities. We wished to evaluate a more real-world scenario in which patients are receiving IT to multiple allergens. Another potential limitation is that we used PBMCs for all our analyses. Using nasal biopsy samples may have yielded more robust findings, in that a nasal biopsy analysis is focused on the specific site of allergic inflammation. This invasive procedure is difficult to perform, especially for the current study which analyzed the same patients sequentially during the first few weeks of their RIT. Nonetheless, we were able to see significant changes in gene expression over the course of RIT by employing PBMC.

In conclusion, this study of allergic rhinitis patients undergoing multi-aeroallergen RIT revealed significant changes in gene expression early after RIT. These studies support a model whereby RIT induces a rapid and potent T helper cell response which alters B cell antibody production resulting in IgG4 which has the potential to bring relief from allergic symptoms.

## List of Abbreviations

*A20 *(or ***TNFAIP3***): tumor necrosis factor, alpha-induced protein 3; **BCL6**: B-cell CLL/lymphoma 6; **BH-FDR**: Benjamini-Hochberg False Discovery Rate multiple testing correction; **BTK**: Bruton's Tyrosine Kinase; ***CA1***: Carbonic Anhydrase I; **CCR**: Chemokine CC-motif Receptors; **CD40L**: CD40 ligand; ***CDC10 ***(or ***SEPT7***): Cell Division Cycle 10; **cDNA**: Complementary DeoxyriboNucleic Acid; ***CLTC***: Clathrin, Heavy Polypeptide; **COX2**: Cyclooxygenase 2; **cRNA**: Complementary Ribonucleic Acid; ***CROP***: Cisplatin Resistance-associated Overexpressed Protein; **CXCL**: Chemokine CXC-motif Ligand; **CXCR**: Chemokine CXC-motif Receptor; ***D***: *Dermatophagoides*; **DUSP1**: Dual Specificity Phosphatase 1; **EEF1B2**: Eukaryotic Translation Elongation Factor 1 beta-2; **FcεRI**: high-affinity IgE receptor-alpha; ***GATA3***: GATA Binding Protein 3; ***IFIT2***: Interferon-induced protein with tetratricopeptide repeats 2; **IFNγ**: Interferon-gamma; ***IFNGR***: Interferon Gamma Receptor; **Ig**: Immunoglobulin (e.g., IgE, IgG); **IL-1β**: Interleukin 1β; ***IL2RB***: Interleukin 2 Receptor, Beta; **IL-8**: Interleukin 8; ***IL13RA1***: Interleukin 13 Receptor, Alpha 1; **IPA**: Ingenuity Pathways Analysis; ***IRF1***: Interferon Regulatory Factor 1; **ISGF-3**: Interferon Stimulated Transcription Factor 3; ***ITGAM ***(or ***CD11b***): Integrin, alpha M (complement component 3 receptor 3 subunit); **IT**: immunotherapy; **kU[A]/L**: kilounits of allergen-specific IgE per liter; **mg [A]/L**: milligram allergen-specific IgG4 per liter; **MX-1**: Myxovirus (Influenza virus) Resistance 1, Interferon-inducible Protein p78; **PBMC**: peripheral blood mononuclear cells; **PBS**: phosphate buffered saline; **PKM2**: Pyruvate Kinase, M2; **RIT**: rush immunotherapy; **RPL9**: Ribosomal Protein 9; **TGF-β**: Transforming Growth Factor beta; **TH**: T Helper cell; **TNF**: tumor necrosis factor; **TRAIL**: TNF Related Apoptosis Inducing Ligand;

## Competing interests

The authors declare that they have no competing interests.

## Authors' contributions

DK, SB, SRB enrolled patients and recorded patient demographics and symptom assessments. DK, SB, SRB and LD collected whole blood for cellular analyses and serum for allergen-specific immunoglobulin levels. SB, SRB and LD assisted with samples for leukocyte counts, prepared total cellular RNA for microarray and carried out phenotyping of PBMC for basophil surface FcεRI and T-regulatory cell frequencies. DK, SB and LD wrote the manuscript. All authors have read and approved the manuscript.

## Supplementary Material

Additional file 1 **Table S1: PBMC display differentially expressed genes during RIT**. An excel (.xls) spreadsheet depicts the expressed genes identified by Ingenuity Pathway Analysis that were derived from the 507 transcripts (≥1.5 fold change) sorted by biological function and including the p-value and a description of the molecule as well as the cellular localization. Click here for file

Additional file 2 **Figure S1: Basophil FcεRI expression is modulated by omalizumab**. Basophil FcεRI expression (gated as Lineage-1^-^, HLA-DR^+ ^and CD123^+^) is displayed as histograms for two experiments (A and B). Isotype control antibody binding is shown for two asthma patients (tinted histogram with gray lines) with similar results for healthy donors (not shown). Basophil FcεRI expression is shown for healthy donors (gray line) as controls and asthma patients on omalizumab for at least 3 months (black line). Click here for file
