# Supplementary figures and images for: Early gene expression changes with rush immunotherapy
Source: Clin Mol Allergy. 2011 Sep 30;9:12. doi: 10.1186/1476-7961-9-12 (PMC3195724; doi:10.1186/1476-7961-9-12)

## Slide 1
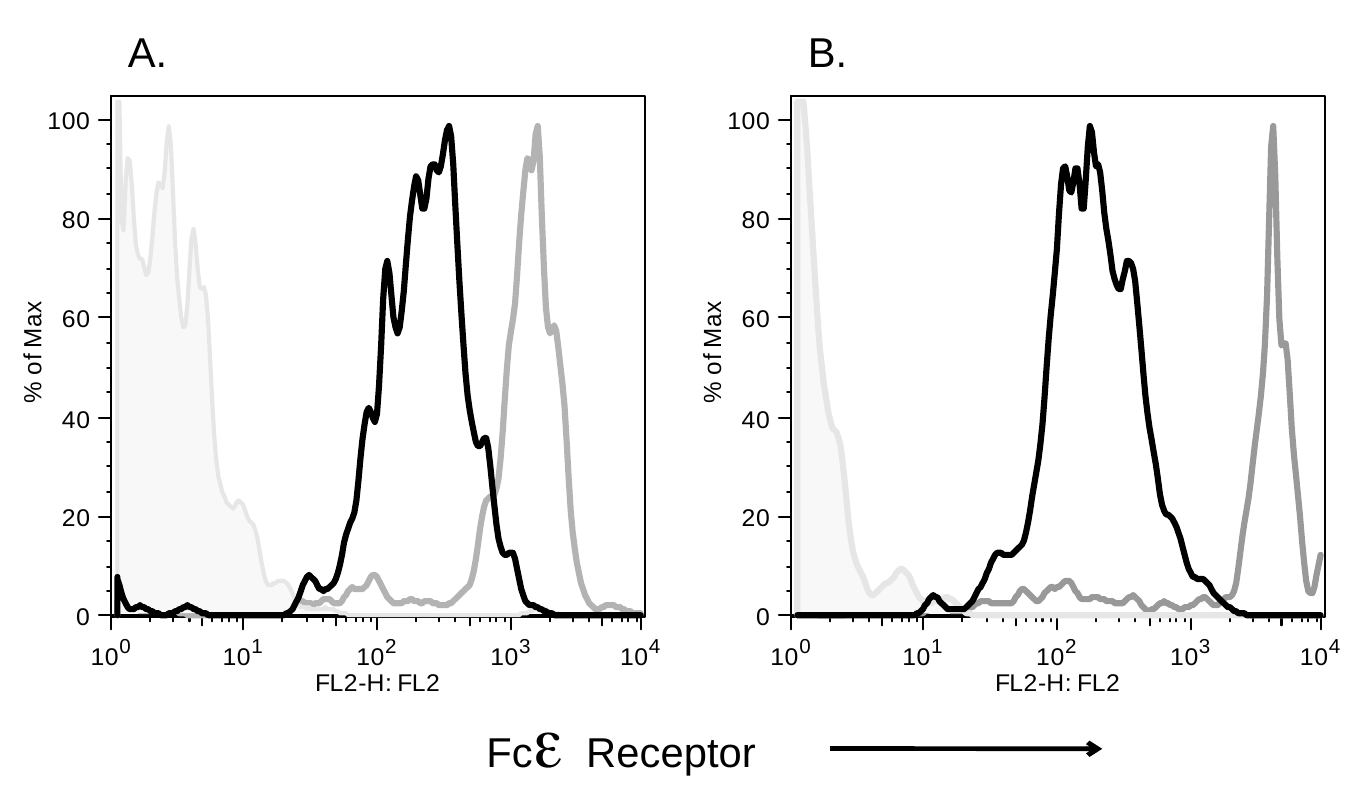

A.
B.
Fcε Receptor

Supplement: Additional file 2 — Figure S1: Basophil FcεRI expression is modulated by omalizumab. Basophil FcεRI expression (gated as Lineage-1-, HLA-DR+ and CD123+) is displayed as histograms for two experiments (A and B). Isotype control antibody binding is shown for two asthma patients (tinted histogram with gray lines) with similar results for healthy donors (not shown). Basophil FcεRI expression is shown for healthy donors (gray line) as controls and asthma patients on omalizumab for at least 3 months (black line). [file 1476-7961-9-12-S2.PPT]
